# The “Hit and Run” Hypothesis for Alzheimer’s Disease Pathogenesis

**DOI:** 10.3390/ijms25063245

**Published:** 2024-03-13

**Authors:** Tal Ganz, Tamir Ben-Hur

**Affiliations:** 1 Faculty of Medicine, Hebrew University of Jerusalem, Jerusalem 91120, Israel; tal.ganz@mail.huji.ac.il; 2 The Department of Neurology, The Agnes Ginges Center for Human Neurogenetics, Hadassah—Hebrew University Medical Center, Jerusalem 91120, Israel

**Keywords:** Alzheimer’s disease, microglia, diabetes mellitus type 2, infections

## Abstract

Alzheimer’s disease (AD) is a devastating neurodegenerative disorder affecting millions worldwide. Emerging research has challenged the conventional notion of a direct correlation between amyloid deposition and neurodegeneration in AD. Recent studies have suggested that amyloid and Tau deposition act as a central nervous system (CNS) innate immune driver event, inducing chronic microglial activation that increases the susceptibility of the AD brain to the neurotoxicity of infectious insults. Although modifiable risk factors account for up to 50% of AD risk, the mechanisms by which they interact with the core process of misfolded protein deposition and neuroinflammation in AD are unclear and require further investigation. This update introduces a novel perspective, suggesting that modifiable risk factors act as external insults that, akin to infectious agents, cause neurodegeneration by inducing recurrent acute neurotoxic microglial activation. This pathological damage occurs in AD pathology-primed regions, creating a “hit and run” mechanism that leaves no discernible pathological trace of the external insult. This model, highlighting microglia as a pivotal player in risk factor-mediated neurodegeneration, offers a new point of view on the complex associations of modifiable risk factors and proteinopathy in AD pathogenesis, which may act in parallel to the thoroughly studied amyloid-driven Tau pathology, and strengthens the therapeutic rationale of combining immune modulation with tight control of risk factor-driven insults.

The amyloid hypothesis posits that the accumulation of beta-amyloid initiates a cascade of events leading directly to progressive neurodegeneration. While this theory has shaped research and therapeutic approaches for AD, multiple studies have highlighted its limitations. There is no correlation between the accumulation of amyloid deposits to clinical deterioration and neuronal loss, and the large temporal gap between amyloid β (Aβ) deposition and neurodegeneration argues strongly against a direct causal relationship [1]. These suggest that additional factors might contribute to AD pathogenesis. Subsequently, the neurodegenerative process has been attributed mainly to the involvement of other misfolded proteins, mainly Tau [2], as well as α-Synuclein and TDP43, with marked pathologic variability [3]. While multiple risk genes contribute to an inherent tendency to develop AD, up to 50% of the risk of developing AD is attributed to modifiable risk factors [4]. Among the systemic risk factors are diabetes mellitus, hypertension, hypercholesterolemia, smoking, and cardiovascular disease. In addition, external insults, such as infectious agents and air pollutants, have been linked to AD pathogenesis. However, it is still unclear how the modifiable risk factors interact with the core pathological process of misfolded protein deposition in AD. Animal studies have suggested that the increased risk attributed to these states is not merely by co-morbidity but by exacerbating AD brain pathology, as indicated by amyloid deposition and gliosis. For example, animal models of diabetes mellitus demonstrated that insulin resistance stimulates the action of β and γ secretases and causes reduced Aβ clearance, leading to its accumulation [5]. Hypertension-induced cerebral ischemia leads to the accumulation of amyloid precursor protein and Aβ [6]. In addition, diet-induced hypercholesterolemia increases Aβ levels, and total Aβ levels correlate strongly with total cholesterol levels in plasma and CNS [7].

However, it is unclear whether modifiable risk factors just accelerate AD pathology or also induce brain injury directly. It is furthermore unclear whether such risk factor-induced injury occurs as a simple addition to misfolded protein-mediated injury or whether a synergistic association occurs between them. Finally, the mechanisms by which risk factors contribute to brain injury and whether different modifiable risk factors share similar mechanisms of injury are not entirely clear. Here, we suggest that modifiable risk factors may act as external insults that cause neurodegeneration, and that risk factor-induced neurodegeneration is enabled particularly by the AD pathology-burdened, highly susceptible, and penetrable brain and is mediated by reactive neurotoxic microglia.

Recent investigations have uncovered the pivotal role of microglial activation in AD. Amyloid β is an antibacterial protein that activates the brain’s innate immune system [8]. Consequently, the amyloid-burdened brain displays widespread activated glial cells releasing large amounts of inflammatory mediators. Cumulating studies have established that neurotoxic microglia can kill neurons [9]. Furthermore, Tau oligomers are also associated with a neuroinflammatory response [10], and Tau-driven neurodegeneration is mediated by immune cells [11]. To mechanistically connect these insights with the contribution of risk factors to AD pathogenesis, we suggest that chronic misfolded-protein-induced glial activation primes the brain for susceptibility to various neurotoxic insults. We suggest that external insults, driven by modifiable risk factors, contribute directly to neurodegeneration through a “hit and run” mechanism. These injurious insults are mediated by the recurrent acute neurotoxic activation of microglia, the “hits”, which is enabled by their baseline-activated state, due to the AD pathology (Figure 1). These insults leave no distinct trace in the brains other than the loss of neurons, “the run”, making it difficult to identify their involvement.

We have recently shown that the presence of AD pathology causes CNS hyper-vulnerability to the neurotoxicity of microbial pathogen-associated molecular patterns (PAMPs). We found that microbial TLR2 and TLR4 agonists kill cortical neurons and that brains inflicted with AD pathology are significantly more vulnerable to their neurotoxicity by two mechanisms [12,13]. First, in transgenic AD mouse models, the compromised blood–brain barrier (BBB) enabled the penetration of systemically administered microbial PAMPs to the CNS. Consequently, we demonstrated that systemically administered PAMPs induce neurodegeneration in 5xFAD mice but not in wild-type (wt) mice. Second, we showed that the direct intra-cerebro-ventricular (ICV) delivery of microbial TLR2 and TLR4 agonists causes cortical neuronal death in a dose-dependent manner and that brains inflicted with AD pathology exhibit a marked increase in cortical neuron death, as compared to wt brains [13]. The increased vulnerability of AD brains to microbial PAMPs was due to the doubling of tissue microglia density and an additional 5-fold increase in the fraction of (neurotoxic) inducible nitric oxide synthase-positive (iNOS+) microglia [13]. Notably, PAMP-induced neurodegeneration was prevalent in cortical regions rich in microglia but not observed in microglia-poor regions, such as the densely packed CA1 and CA3 of the hippocampus [12]. In addition, we showed that either the depletion of microglia or modulation of the microglial neurotoxic phenotype by the direct ICV delivery of a retinoic acid receptor α agonist [14] prevents microbial PAMP-induced neurodegeneration [12,13]. Altogether, these suggest that the neurotoxic activation of microglia by microbial PAMPs mediates neurodegeneration and that amyloid-induced microglial priming followed by neurotoxic activation is reversible, offering potential avenues for therapeutic intervention.

Building on these findings, we suggest a revised immune-mediated model for neurodegeneration in AD, where systemic risk factors act as external neurotoxic insults in the amyloid- and Tau-burdened AD brain. In this model, amyloid and Tau deposition serves as the “initiating” event, triggering chronic microglial activation. This sustained activation renders the brain susceptible to various neurotoxic insults, both internal and external. These insults may induce acute neurotoxic microglial activation through a “hit and run” mechanism, leaving no lasting pathological trace in the brain, other than a loss of neurons. As neurodegeneration is predominantly observed in areas rich in misfolded proteins and microglia, it is challenging to directly prove an insult’s contribution to acute microglial activation, which leads to neuronal loss and contributes to AD progression.

The above-described mechanism, shown in animal models, may explain the clinical observations of long-lasting cognitive decline in AD patients following systemic infections and the linkage between specific infectious agents and AD occurrence and progression [15,16,17]. Several oral pathogens causing periodontitis, such as Treponema denticola and Porphyromonas gingivalis, have been implicated in AD pathogenesis. However, it seems that mainly general mechanisms of systemic and CNS inflammation underlie this association, converging to microglia [18,19,20]. To further elucidate the relevance of the “hit and run” mechanism in AD pathogenesis, it is important to examine the effect of various external insults on glial activation and neurodegeneration, beyond infections. We discuss this mechanism regarding two cardiovascular/metabolic risk factors of AD, namely diabetes mellitus type 2 (T2D) and hypercholesterolemia.

T2D is considered one of the major risk factors for AD, doubling its risk [21]. However, T2D does not increase the amount of amyloid or Tau pathology nor of PET or CSF biomarkers of amyloid pathology [22,23,24]. Instead, several clinical observations support the notion that T2D acts as an external insult, causing neurodegeneration in AD directly, as indicated by several imaging parameters [25]. These suggest that T2D accelerates neurodegeneration without increasing the burden of AD core pathology. Moreover, glycemic variability has emerged as an independent factor in predicting diabetic complications, including retinal neurodegeneration [26]. In terms of cognitive performance, hyperglycemic events are associated with acute cognitive dysfunction [27], and the increased occurrence of hyperglycemic events in T2D is associated with chronic cognitive impairment [28]. Improved glycemic control both delayed and slowed the rate of cognitive decline [29]. Finally, the cognitive declines in patients with diabetes mellitus were mediated by neurodegeneration [30,31]. The effect of T2D on cognitive decline and neurodegeneration may be mediated by multiple metabolic causes, oxidative stress, and the activation of neuroinflammation [32,33]. Thus, glycemic variability, along with other effects of persistent hyperglycemia, may act on a pre-conditioned and vulnerable brain to accelerate neurodegeneration. Given the chronic gliosis and activation of microglia triggered by AD pathology, uncontrolled T2D may cause recurrent neurotoxic microglial activations, which mediate cortical neuronal loss via a “hit and run” mechanism. Indeed, multiple lines of evidence have shown that diabetes activates microglia, as indicated by metabolic reprogramming, the upregulation of markers of activity and cellular inflammatory pathways, morphological changes, the upsurge in the production of pro-inflammatory cytokines, and increased oxidative stress [34,35]. Diabetes induces neurotoxic microglial activation by several mechanisms. First, mitochondrial dysfunction has been extensively reported in patients with either AD or diabetes. This dysfunction serves as a pivotal catalyst for inflammasome formation, ultimately resulting in neuronal damage [36]. Another mechanism by which T2D contributes to microglial activation is through BBB malfunction and the activation of peripheral immune cells releasing pro-inflammatory cytokines which lead to central inflammation [36,37]. Finally, there is growing evidence that, in agreement with clinical observations, glycemic variability itself significantly drives microglial polarization to an inflammatory phenotype [38], along with increased oxidative stress, leading to neuroinflammation and cognitive dysfunction [39].

In addition, accumulating evidence suggests a link between cholesterol metabolism and AD progression. Specifically, 7-ketocholesterol (7KC), a product of cholesterol interaction with oxygen radicals, activates microglia. This compound triggers microglial proliferation, migration, and classical M1 activation, producing inflammatory factors [40]. This and other cholesterol oxides confer a neurotoxic microglial phenotype, which induces programmed cell death by increased nitric oxide production and potentiated LPS effects [41]. In agreement, microgliosis-associated memory deficits, increased apoptosis (without a change in brain amyloid-β levels), and increased susceptibility to amyloid-β-induced neurotoxicity (a potent microglial activator in itself) were observed in a hypercholesterolemic mouse model [42,43,44]. A recent study found that not only high blood lipids in themselves but also their fluctuations in time are associated with an increased risk of developing AD [45], suggesting that both chronic and acute cholesterol-mediated insults might induce brain injury, in accordance with the “hit and run” process.

This hypothesis, positing microglial activation as a pivotal player in neurodegeneration following modifiable risk factor-mediated insults, necessitates further investigation. We need to continue learning and establish the following: (1) which systemic risk factors act as insults causing neurodegeneration in the susceptible AD pathology-inflicted brain; (2) whether these risk factors induce neurodegeneration by activating neurotoxic microglia; and (3) whether microglial modulation may be targeted in a unifying manner to mitigate risk factor-induced neurodegeneration. In the realm of clinical trials, microglia have emerged as a potential therapeutic target, and a large fraction of current clinical trials in AD are directed against the neuroinflammatory process [46]. However, current approaches predominantly focus on inhibiting microglial activity without addressing the need for their modulation toward a neuroprotective state. Hence, we emphasize the need to modulate microglia towards a neuroprotective polarized state rather than mere inhibition as a possible therapeutic strategy for AD, combined with prophylactic treatments that reduce the severity of systemic insults. The rationale is that the modulation of microglia may be effective in protecting the brain, only if combined with the tight regulation of those systemic risk factors which induce their neurotoxic activation. Importantly, we need to identify which of the various risk factors, previously considered independent contributors to disease progression, may converge through common pathways involving the neurotoxic activation of microglia. The categorization of patients according to microglial-mediated, risk factor-induced tissue injury (as compared to other risk factor mechanisms of injury) will increase the likelihood of successful drug development for AD prevention by modulating microglial neurotoxicity and enable patient-tailored preventive therapies. For example, some anti-diabetic medications, such as metformin and dipeptidyl peptidase-4 inhibitors, also possess microglial-modulatory properties [47,48] and may therefore be suitable for risk factor management and neuroprotection by immune modulation.

In conclusion, the revised immune-mediated hypothesis connects amyloid and Tau deposition, microglial activation, and the brain’s susceptibility to systemic risk factors that may act as neurotoxic insults. This viewpoint sheds light on the complex pathogenesis of AD and highlights the modulation of the immune response and mitigation of external insults as a potentially effective strategy for treating/preventing AD. Continued research is crucial to unravel the intricate mechanisms involved in AD development and bring us closer to finding a cure for this devastating disease.

## Figures and Tables

**Figure 1:**
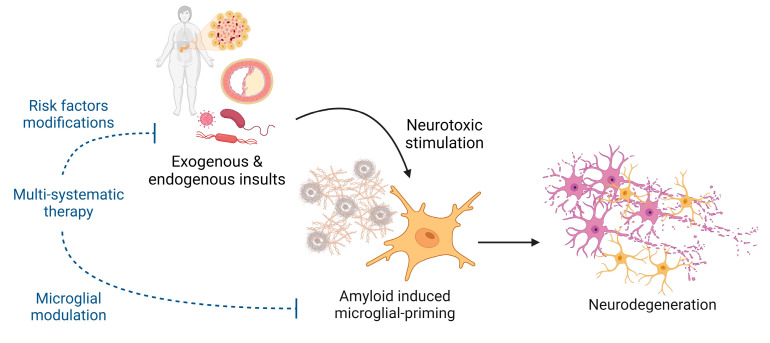
Neurodegeneration is caused by the neurotoxicity of both internal and external insults, including metabolic risk factors, infections and infectious particles (pathogen-associated molecular patterns), air pollutants, and others, acting on the hyper-vulnerable brain displaying the Alzheimer’s disease pathology. Created with www.BioRender.com.
